# Ultrasound-guided suprainguinal fascia iliaca block combined with a sacral plexus block for lower extremity surgery: A case report

**DOI:** 10.1097/MD.0000000000021921

**Published:** 2020-08-28

**Authors:** Jie Zhao, Yanming Huang, Mingjie Fu, Fan Tao

**Affiliations:** a Hangzhou Red Cross Hospital, Anesthesiology Department; b Surgical Intensive Care Unit, First Affiliated Hospital of Zhejiang University, School of Medicine, Hangzhou, China.

**Keywords:** lower extremity surgery, sacral plexus block, suprainguinal fascia iliaca block

## Abstract

**Rationale:**

The anesthetic management of patients with severe pulmonary hypertension is different from that of normal, healthy patients, and regional nerve blocks are commonly used for them. Due to the individual variability of the course, distribution, and branching of the nerves below the inguinal ligament, the supra-inguinal fascia iliaca (SIFI) block has a wider and more stable blocking area. In combination with the sacral plexus block, they can satisfy the needs of surgical anesthesia below the hip.

**Patient concerns:**

A 46-year-old man with tuberculosis, chronic obstructive pulmonary disease, pulmonary heart disease, World Health Organization (WHO) class III pulmonary hypertension and right heart dysfunction, and American Society of Anesthesiologists physical status class III needed fixation of an intramedullary nail in the left lower extremity. Additionally, he had broken his left lower limb after a recent fall. Both general anesthesia and epidural anesthesia were not appropriate.

**Diagnoses:**

The patient had a clear history of tuberculosis, computerized tomography scan displayed destructive pneumonophthisis. Furthermore, he had chronic obstructive pulmonary disease and pulmonary heart disease.

**Interventions:**

An ultrasound-guided SIFI combined with a sacral plexus block was successfully performed for surgical anesthesia and avoided all hemodynamic fluctuations.

**Outcomes:**

We successfully performed an ultrasound-guided SIFI combined with a sacral plexus block for surgical anesthesia and avoided all hemodynamic fluctuations.

**Lessons:**

Ultrasound-guided suprainguinal fascia iliaca block combined with a sacral plexus block can be suitable for anesthesia for patients with severe circulatory compromise.

## 1 Introduction

The choice of anesthesia for patients with severe pulmonary hypertension is not limited to traditional techniques. Peripheral nerve blocks have specific advantages over general anesthesia as they are relatively simpler, have a high safety profile, is exact in their effects, and have little physiological interference. In recent years, it has been increasingly used for anesthesia in the elderly, especially in critically ill patients. Multimodal analgesia with nerve blocks have been recommended in the perioperative period of lower extremity joint arthroplasty.^[1]^ Fascia iliaca compartment block (FICB) is commonly used to provide anesthesia and analgesia in hip replacement surgeries.^[2,3]^ Due to the individual variability in the course, distribution and branching of nerves below the inguinal ligament, the FICB used to block the lateral femoral cutaneous nerve (LFCN) has a reported failure rate of 10% to 37%.^[4]^ However, above the inguinal ligament, it has a reliable course immediately below the fascia iliaca in the pelvis. Therefore, an improved FICB named the suprainguinal fascia iliaca (SIFI) block that is a viable alternative to the traditional fascia iliaca techniques and to the femoral nerve block was developed.^[5]^ As an improved nerve block, the SIFI combined with the sacral plexus block has not been widely used in clinical anesthesia. We present the case report of a patient in whom an ultrasound-guided SIFI combined with sacral plexus block achieved good anesthetic results. Written consent was obtained from the patient for the publication of this report.

## 2 Case report

**Figure 1:**
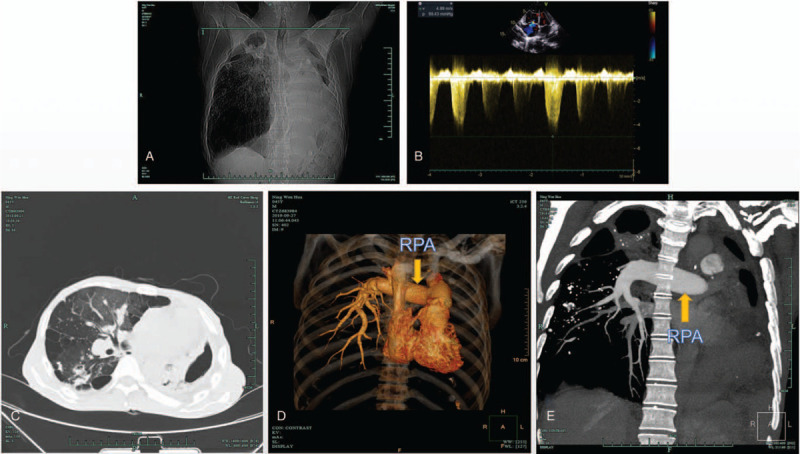
Patient imaging data. A and C: Chest x-ray and Computed Tomography (CT) scan indicated severe tuberculous damage in left lung and compensatory hyperventilation in the right lung, also with diffuse lesions. B: Echocardiography, based on tricuspid regurgitation, estimated pulmonary artery systolic pressure as high as 109 mm Hg. D and E: Pulmonary Computed Tomographic Angiography (CTA) and vascular reconstruction showed that the right pulmonary artery (RPA) was significantly widened (>30 mm), while the left pulmonary artery was not developed.

**Figure 2:**
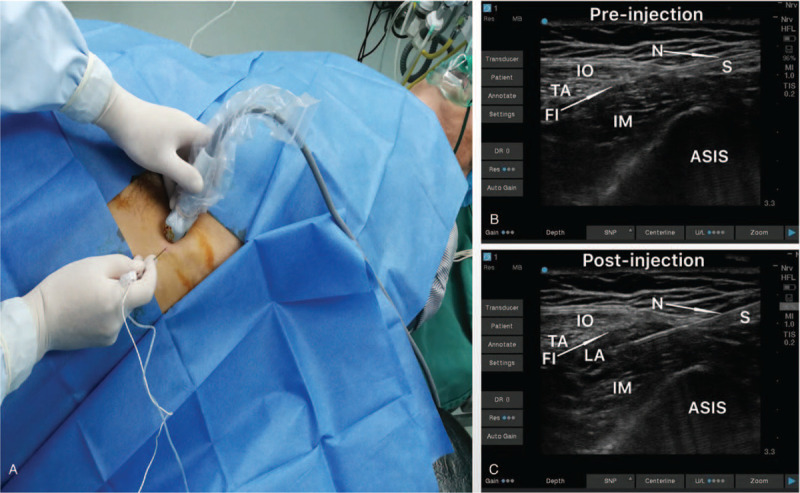
Ultrasound-guided suprainguinal fascia iliaca block. A: Final ultrasound position with lateral aspect of probe superior to ASIS and medial aspect rotated to point at perineum. Needle was entering at center of probe in an in-of-plane fashion. B: Pre-injection ultrasound image of ASIS, internal oblique muscle, transverse abdominus muscle, and iliacus muscle. C: Post-injection ultrasound image showing local anesthetic deposition immediately beneath the fascia iliaca. ASIS = anterior superior iliac spine; FI = fascia iliaca; IM = iliacus muscle; IO = internal oblique muscle; LA = local anesthetic; N = needle; S = Sartorius; TA = transverse abdominus.

The patient was a 46-year-old man who presented with pain (2–4 NRS scores) and difficulty in moving his left lower limb after a fall. He was diagnosed with a left femoral neck fracture, and surgery for the fixation of the left lower extremity with an intramedullary nail was planned. Preoperative interview revealed a history of tuberculosis and a subsequent pulmonary CT revealed severe tuberculous damage in left lung and diffuse lesions in right lung (Fig. 1). Echocardiography showed significant pulmonary arterial hypertension (109 mm Hg). The patient was classified as American Society of Anesthesiologists (ASA) class III. Ultimately, we performed an ultrasound-guided SIFI block combined with a sacral plexus block. After obtaining preoperative informed consent, sedation was initiated using an infusion of dexmedetomidine (1 μg/kg maintenance). Using all aseptic precautions, the anterior superior iliac spine (ASIS) was first palpated and a high frequency linear ultrasound transducer (UMT-400, Mindray, China) was placed over the inguinal ligament to identify the femoral artery. The probe was then moved laterally to identify the sartorius muscle and to place it at the center of the ultrasound field of vision. The probe was then rotated 30°, just perpendicular to inguinal ligament and moved towards the femoral head to identify the internal oblique muscle, iliacus muscle, and the ilium (Fig. 2). Local anesthesia of puncture site was performed with 5 mL of 1% lidocaine, and a continuous plexus block needle (1000 mm × 20 G, Germany) for catheter placement was advanced in an in-plane technique, in the cephalad direction, to enter the superficial part of the fascia iliaca. The position of the needle tip was confirmed by injection of 2 mL of normal saline, and its depth was adjusted according to the diffusion of the solution. After reaching the target position, 0.375% ropivacaine was injected (20 mL), and the diffusion of the solution between the iliac fascia and iliac muscle was clearly noticed during the injection (Fig. 2). After the drug took effect (about 3 minutes), the tactile and temperature sensations of the blocked and non-blocked sides were tested by ice water. After the SIFI block, his pain was relieved significantly, and we proceeded to perform the next block.

**Figure 3:**
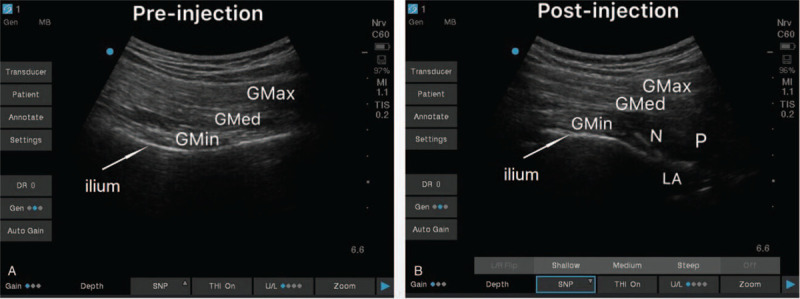
Ultrasound-guided sacral plexus block. A: Pre-injection ultrasound image of gluteus maximus, gluteus medius, gluteus minimus, and ilium. B: Post-injection ultrasound image showing local anesthetic between ilium and piriformis. GMax = gluteus maximus; GMed = gluteus medius; GMin = gluteus minimus; N = needle; P = piriformis.

The sacral plexus block was performed under all aseptic precautions in the left lateral decubitus position. The ultrasonic convex array probe was placed at the midpoint of the line between the greater trochanter of the femur and posterior superior iliac spine. The long axis of the ultrasound probe was placed perpendicular to the spine. As the probe was moved inward and downward, the ilium separated on the image, and the sacral plexus was seen as the highlighted (Fig. 3) oval below the ilium. After injecting 5 mL of 1% lidocaine at the skin puncture site, the block needle was advanced in an in-plane technique, to reach the sacral plexus and 20 mL of 0.375% ropivacaine was injected.

The surgery lasted 45 minutes and no additional opioids were given, except for 0.05 μg/kg of intravenous fentanyl before the skin incisions. The patient was comfortable and maintained stable vital signs during the operation. The patient was discharged from the hospital on the fifth postoperative day without any complication.

## 3 Discussion

Epidural anesthesia, which is often used for hip and lower extremity surgery, has a quick onset and reliable anesthetic effect. However, due to the blockage of the sympathetic preganglionic fibers, it results in obvious hemodynamic fluctuations. On the other hand, accurate regional nerve block can achieve anesthesia with minimal impact on the human body. Due to the advantage of anatomy, regional nerve blocks are more suitable for anesthesia and analgesia for surgical procedures on the extremities.^[6]^

Since the SIFI block can block the LFCN more successfully, it appears to be a viable alternative to the traditional fascia iliaca block techniques and to the femoral nerve block, both of which have been used to aid postoperative analgesia following total hip arthroplasty.^[5]^ The SIFI block anesthetizes a wider anatomical area and can be performed with the patient in the supine position, thereby avoiding the need to move them while they are still in pain due to their fractures. The sacral plexus is mainly composed of the anterior branches of the L4–5, S1–5, and C01 spinal nerves. The main branches of the sacral plexus are the superior gluteal nerve, inferior gluteal nerve, pudendal nerve, posterior cutaneous nerve, sciatic nerve, quadratus femoris nerve, and obturator internus nerve.^[7]^ Ben-Ari et al^[8]^ successfully located the sacral plexus by ultrasound in 2009, which provided the theoretical basis for ultrasound-guided sacral plexus blocks. In 2012, Pearce^[9]^ and Taha^[10]^ described the ultrasound localization technique of the sacral plexus block in detail. There are 2 approaches for the sacral plexus block currently: the posterior ultrasound guided sacral plexus block technique and lateral ultrasound guided sacral plexus block technique.^[11]^ The former provides clearer visualization of the anatomical structures under ultrasound, while the latter is more suitable for patients who cannot lie on their side.

When the SIFI block is combined with the sacral plexus block, almost the whole area of buttock, perineum and limb can be blocked, which can satisfy the anesthetic requirements for surgeries of the caput femoris and hip. At the same time, nerve blocks have fewer complications, such as hematoma at the puncture site and hemodynamic instability, and therefore, it has no effect on the administration of anticoagulant drugs after orthopedic surgery. Simultaneously, since peripheral nerve block does not block pelvic and abdominal visceral nerves, the patients will not have urinary retention, nausea, or vomiting after surgery. Additionally there is no need for postoperative fasting, and no risk of spinal headache.^[12]^ Further, nerve blocks can provide adequate postoperative analgesia, enabling the patients to get out of bed early for routine activities and exercises to strengthen the joint function.

In summary, this compound nerve block is more suitable for elderly patients with severe cardiopulmonary insufficiency or unstable circulation. The only limitation would be the expertise required by the anesthetist to gain sufficient proficiency in performing these ultrasound guided blocks.

The role of contemporary anesthesia is not only to ensure that the surgery is conducted smoothly, but also to give consideration to the patient's comfort, and even the individual's anesthetic choice. In the next step, we will apply the SIFI block combined with sacral plexus block to those with severe circulatory disturbances, and observe the effects on hemodynamic fluctuations, as well as the incidence of postoperative complications. Prospective studies of efficacy are now being planned.

## Author contributions

**Jie Zhao:** This author developed this manuscript.

**Mingjie Fu:** This author helped patient care postoperatively.

**Yanming Huang & Fan Tao:** This author helped ultrasound-guided operations.
